# Insights into Host Cell Modulation and Induction of New Cells by the Corn Smut *Ustilago maydis*

**DOI:** 10.3389/fpls.2017.00899

**Published:** 2017-05-29

**Authors:** Amey Redkar, Alexandra Matei, Gunther Doehlemann

**Affiliations:** ^1^The Sainsbury Laboratory, Norwich Research ParkNorwich, United Kingdom; ^2^Botanical Institute and Cluster of Excellence on Plant Sciences, University of Cologne, BiocenterCologne, Germany

**Keywords:** *Ustilago maydis*, tumor, cell-specific, induction, maize

## Abstract

Many filamentous fungal pathogens induce drastic modulation of host cells causing abnormal infectious structures such as galls, or tumors that arise as a result of re-programming in the original developmental cell fate of a colonized host cell. Developmental consequences occur predominantly with biotrophic phytopathogens. This suggests that these host structures result as an outcome of efficient defense suppression and intimate fungal–host interaction to suit the pathogen’s needs for completion of its infection cycle. This mini-review mainly summarizes host cell re-programming that occurs in the *Ustilago maydis* – maize interaction, in which the pathogen deploys cell-type specific effector proteins with varying activities. The fungus senses the physiological status and identity of colonized host cells and re-directs the endogenous developmental program of its host. The disturbance of host cell physiology and cell fate leads to novel cell shapes, increased cell size, and/or the number of host cells. We particularly highlight the strategies of *U. maydis* to induce physiologically varied host organs to form the characteristic tumors in both vegetative and floral parts of maize.

## Introduction

Many fungal plant pathogens cause drastic morphological changes in colonized host organs. Symptoms arising from these interactions often lead to the formation of galls and tumors, which result from hypertrophy and hyperplasia after activation of host cell proliferation and excessive growth (Wildermuth, 2010). On the other hand, morphological variations could also arise by growth suppression. One example is the suppression of internodal growth that may give rise to the witches’ broom phenotype resulting in stunted growth and loss of apical dominance in the host plant as observed after basidiomycete *Moniliophthora perniciosa* infection of *Theobroma cacao* (Teixeira et al., 2014). Another recognized example in this context is the *Gibberella* species complex some of which cause bakanae disease of rice resulting from cell expansion due to a surplus in the phytohormone gibberellic acid (Desjardins, 2003). Mechanisms for generation of the novel plant structures depend on the pathogen’s lifestyle, but commonly involve host cell re-programing by defense suppression, transcriptional regulation, re-direction of nutrient fluxes, and perturbation of metabolic pathways.

Mainly basidiomycete pathogens induce enlargement and de-differentiation of colonized tissues leading to prominent symptoms. Examples include *Gymnosporangium juniper-virginianae* that causes the cedar apple rust disease, which is named “yellow slender monster” and is mainly thought to involve pathogen-produced Indole Acetic Acid (IAA) (Agrios, 2005). Some wild species of *Acacia* are also infected by gall forming rusts. Examples are *Atelocauda digitate* causing gall rust disease (Nelson, 2009), and *Ravenelia esculenta* in which thorns and the young apical meristems are transformed into hypertrophied tissues that arise from pathogen interference with host auxin activity (Kuvalekar et al., 2008). Some rust fungi can cause galls on their colonized host, i.e., *Uromyces hobsonii* that induces galls on all aerial parts of the aromatic oil plant *Jasminum officinale* var *grandiflorum* (Kuvalekar et al., 2011). The smut fungi (*Ustilaginales*) also cause strong changes in tissue morphology upon host infection, both before and during sporulation (Luttrell, 1981). Apart from stunting, many infected plants are virtually symptomless until the fungus begins to sporulate (Luttrell, 1981). Exceptions from this infection style include *Ustilago esculenta*, a pathogen that causes stem galls on *Zizania latifolia* resulting from pathogen-produced IAA and cytokinins (Chung and Tzeng, 2004). *Ustilago maydis* causes tumors on all aerial parts of maize. This fungus, which provokes developmental re-programming of both vegetative and floral organs, does not directly produce plant hormones. In this mini-review, we summarize recent developments related to the modulation of organ development and host cell differentiation in the *U. maydis* – maize interaction.

## *Ustilago maydis*: A Model to Study Cell Re-Programming in Biotic Interactions

*Ustilago maydis* is a well-established model fungal pathogen that serves to dissect host cell modulation in a biotrophic interaction (Brefort et al., 2009; Matei and Doehlemann, 2016). *U. maydis* shows a bi-phasic life style with a non-pathogenic phase with yeast-like growth of haploid cells, termed sporidia. Plant infection starts with the formation of a dikaryotic filament that results from the fusion of two compatible sporidia (Kahmann et al., 1995). Colonization of maize begins with formation of a terminal swelling of the dikaryotic filament (termed appressorium), which initiates penetration of the epidermal cells (Snetselaar and Mims, 1993; Lanver et al., 2014). The infectious hyphae establish an extensive biotrophic interaction zone after invagination of the plant plasma membrane. This so-called biotrophic interface constitutes the major interaction site for dealing with initial suppression of plant defense and also for nutrient acquisition. *U. maydis* efficiently suppresses the plant’s innate immune system and manipulates host metabolism, by secreting several hundreds of effectors into the biotrophic interface (Djamei and Kahmann, 2012; Lo Presti et al., 2015). Analysis of the *U. maydis* genome defined more than 700 candidate effector proteins, including proteins that are predicted to be unconventionally secreted (Kämper et al., 2006; Dutheil et al., 2016). Interestingly, 20% of these secreted proteins are arranged in 22 gene clusters, many of which encode effectors and show elevated expression in biotrophic stages (Kämper et al., 2006; Brefort et al., 2014). Several *U. maydis* effectors promoting virulence have been functionally characterized. Pep1 acts in the suppression of the plant oxidative burst by inhibiting the apoplastic plant peroxidase POX12 (Hemetsberger et al., 2012, 2015), and Pit2 inhibits a group of apoplastic cysteine proteases (Mueller et al., 2013). These two effectors play an important role in apoplastic defense suppression early in infection (Doehlemann et al., 2009, 2011). Translocated effectors in *U. maydis* include Cmu1 that suppresses salicylic acid synthesis by regulation of chorismate homeostasis (Djamei et al., 2011), and Tin2, which is involved in stabilizing a maize kinase promoting anthocyanin biosynthesis and resulting in reduced lignin biosynthesis (Tanaka et al., 2014). The translocated effector See1 is required for activation of plant DNA synthesis during tumor formation in maize leaves (Redkar et al., 2015a).

After initial establishment of biotrophy, *U. maydis* manipulates the host leaf primary and secondary metabolism to obtain plant resources for its own growth benefit (Doehlemann et al., 2008). This in turn leads to tumor formation, which is a particular hallmark in *U. maydis* infection that is uncommon in most monocot smut fungi. Extensive research on this pathosystem has focused on early development steps in pathogen establishment and the genetic requirements for pathogenicity (Mendoza-Mendoza et al., 2009; Lanver et al., 2014). The cytology of tumor generation at the later stages during infection is mainly restricted to electron micrographs of infected seedling leaves (Callow and Ling, 1973; Callow, 1975; Snetselaar and Mims, 1992). Interestingly, *U. maydis* has evolved effectors tailored to individual host organs making it a specialized biotroph compared to the closely related smuts *Sporisorium reilianum* or *Ustilago hordei* (Schirawski et al., 2010; Laurie et al., 2012). A transcriptome analysis of *U. maydis* infecting either vegetative or floral tissue revealed that nearly 45% of the genes encoding secretory/secreted proteins in *U. maydis* show an organ-specific expression pattern (Skibbe et al., 2010).

Employing maize developmental mutants a few host requirements for tumor formation have been defined (Walbot and Skibbe, 2010). Additionally, intrinsic mutations in maize were found to disrupt fungal development and can enhance or suppress tumor growth (Walbot and Skibbe, 2010). For example, the male-sterile mutants of maize *msca1* and *mac1* (Sheridan et al., 1999; Chaubal et al., 2003) that are disrupted in proliferative cell types early in anther development, lack *U. maydis* induced tumor formation (Walbot and Skibbe, 2010). On the other hand, the *Kn1* dominant maize mutant (Smith et al., 1992) develops enlarged tumors from ectopic growth of rapidly expanding cells. The *Kn1* mutant is disrupted in a KNOX transcription factor that causes a defect in the restriction of the meristematic tissue which creates a hotspot for *U. maydis* infection. Additionally, several gibberellic acid related maize dwarf mutants exhibit organ-specific resistance to *U. maydis* (Skibbe et al., 2010). This indicates that a normal developmental program is crucial for *U. maydis* to trigger tumor formation by the host. In turn, for the pathogen it is crucial to tailor its virulence proteins to the specific host tissues. Besides tissue-specific adaptation, virulence patterns of *U. maydis* also seem to depend on variety-specific maize factors, which might reflect involvement of a group of variety-specific effectors being expressed by the pathogen. For example, deletion of the *U. maydis* ApB73 effector was recently shown to cause a cultivar-specific loss of tumor formation (Stirnberg and Djamei, 2016).

Combining transcriptome data with functional genetics demonstrated organ-specific virulence functions of individual *U. maydis* effectors (Schilling et al., 2014). A set of seven organ-specific effectors was found to be evolved for tumor formation in vegetative tissues, specifically in leaves (Schilling et al., 2014). On the other hand, only two effectors were found to exclusively contribute to tumor development in tassels (Schilling et al., 2014). This supports the previous finding that a much higher number of effector genes are specifically upregulated during leaf infection, while most effectors active in tassel tumors were also expressed in other plant organs (Skibbe et al., 2010). Together, in light of these observations one might conclude that floral tumors may result from an evolutionary basal set of virulence factors, conserved amongst smuts within the order *Ustilaginales*. Barley infected by *U. hordei* shows floral symptoms in which seeds are replaced by spores during floral development. *S. reilianum* exhibits an intermediate phenotype between *U. maydis* and *U. hordei* showing phyllody by promoting the outgrowth of subapical leafy inflorescence and floral symptoms (Ghareeb et al., 2011). *U. maydis* organ-specific effectors additionally regulate the disease through their qualitative expression in the desired target organ showing transcriptional plasticity and therefore increasing the pathogen’s fitness to different tissue environments (Skibbe et al., 2010; Schilling et al., 2014). Among the leaf-induced genes *um01829* encodes an α-L-arabinofuranosidase (Schilling et al., 2014), which is predicted to degrade (arabino-) xylan from cell walls (Rahman et al., 2003; Numan and Bhosle, 2006). Arabino-xylans constitute one of the most abundant cell wall polysaccharides in maize (Santiago et al., 2013). This secreted pathogen protein may have a role in cell wall loosening to establish cell wall re-formation. Detailed analysis of the cell wall composition in different maize organs could elucidate the reason for the leaf-specific role of this effector.

Of the previously identified set of leaf-specific effector genes, one has been characterized on the functional level of the encoded effector protein. This effector, See1, was found to be required for activation of DNA synthesis during tumor formation (Redkar et al., 2015a).

## Induction of new Cells by *U. maydis* in Maize

**FIGURE 1:**
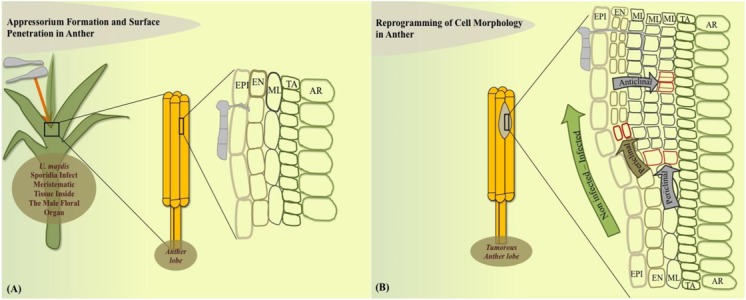
Model of cell-type specific changes upon *Ustilago maydis* redirected maize anther tumor formation. **(A)** Fungal sporidia infect immature anther meristem embedded in leaf whorl. **(B)** Cell morphology is redirected in the dividing anther by initiation of additional periclinal divisions in endothecium and frequent anticlinal and periclinal divisions in the middle layer adds an additional cell layer during the initial stage of tumorigenesis. EPI, epidermis; EN, endothecium; ML, middle layer; TA, tapetum; AR, archesporial cells.

How exactly a tumor is initiated in anatomically varied plant parts has been an active question for several years. Approaches to answer this question have shed light on many interesting host cellular and metabolic modulations that *U. maydis* generates to initiate a tumor. On the cellular level in maize anthers, *U. maydis* can only colonize the immature not yet differentiated anther cells, which retain meristematic activity (Gao et al., 2013; **Figure 1A**). *U. maydis* hijacks this primordial cell stage to reprogram cell fate towards tumor formation in maize anthers. In this organ, it activates the tumor pathway only by redirecting intrinsic cell proliferation, without an oncogenic activity. After 2 days post infection (dpi), the fungus invades the sub-epidermal cells to alter cell fate specification events, ongoing cell division patterns, and cell expansion depending on the anther developmental stage and cell-type. Tumor formation in maize anthers results from ectopic periclinal divisions directed by the fungus in anther somatic cells, initially generating an extra cell layer that results in disrupted anther lobe architecture (**Figures 1A,B**). The most frequent additional anticlinal and periclinal divisions are observed in the middle layer (ML) of infected anthers. ML cells typically undergo only a few anticlinal divisions prior to programmed cell death. Hence, in male floral tissues of maize, *U. maydis* reprograms cell fate of ML cells but does not act as a potent general inducer of cell division (Gao et al., 2013).

**FIGURE 2:**
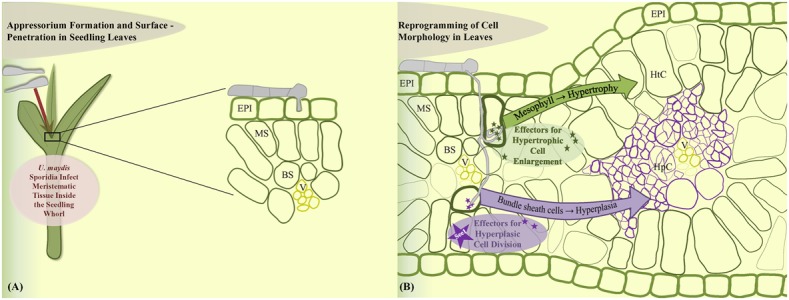
Model of leaf cell-type specific changes upon *U. maydis* induced tumor formation. **(A)** Fungal sporidia induce leaf tumors when the inoculum infects the young developing leaf tissue within the leaf whorl. **(B)** Cell morphology is reprogrammed towards hypertrophy in the mesophyll cells and hyperplasia in the bundle sheath cells. EPI, epidermis; MS, mesophyll; BS, bundle sheath; V, vascular; HtC, hypertrophic cells; HpC, hyperplasic cells.

In contrast, leaf tumors result from a different phenomenon. Maize leaf development represents a linear gradient of cell division wherein most cell divisions occur in a narrow zone at the base of the blade adjacent to the ligule (Li et al., 2010). *U. maydis* infections result from the profuse and rapid cell division in the sub-epidermal leaf cells that are already differentiated. *U. maydis*-induced tumor formation in leaves is initiated on the cellular level around 4 dpi and requires the local presence of fungal hyphae in the zone of tumor development (Banuett and Herskowitz, 1996; Redkar et al., 2015a). Leaf tumor formation is accompanied by cell enlargement as well as cell division (Callow and Ling, 1973; Banuett and Herskowitz, 1996). Plant cells were described to increase in size upon tumor maturation and fungal cells proliferate in this zone and build out aggregates surrounded by a mucilaginous layer larger than neighboring plant cells (Snetselaar and Mims, 1994; Banuett and Herskowitz, 1996; Doehlemann et al., 2008; Tollot et al., 2016). Exact information on how leaf tissue changes at the cellular level as well as knowledge of the cellular origin of tumor cells was lacking. A recent cytological analysis has addressed these issues and showed that development of a leaf tumor involves major cell morphological rearrangements (Matei et al., under review). In summary, leaf tumors result from two distinct cellular processes. Bundle sheath cells undergo hyperplasic cell division to generate novel tumor cells, while mesophyll cells transform into hypertrophic tumor cells (Matei et al., under review; **Figures 2A,B**). Transcriptome analysis of mesophyll vs. bundle sheath-derived cells in incipient tumors revealed cell-type specific effector sets (Matei et al., under review). Identification of the exact role of these cell-type specific virulence factors will constitute a major part of future research. One hypothetical model would propose that development of hypertrophic cells might ensure fungal nutrition while hyperplasic cell division would maintain a sink signal for the attraction of nutrient flow from source tissue.

An important role in the leaf tumor formation is hold by the leaf-specific effector See1. This effector protein was found to be involved in the activation of DNA synthesis in the post-differentiated maize leaf cells, which then divert to a tumorous pathway (Redkar et al., 2015a). See1 is not required in immature maize floral cells and these proliferative cells are simply redirected into the tumor pathway. See1 targets maize SGT1 (suppressor of G2 allele of Skp1), a known cell cycle and immune response modulator (Shirasu, 2009). See1 interferes with defense-induced phosphorylation of SGT1 to prevent activation of downstream immune responses (Redkar et al., 2015a). Cytological analysis of leaf tumor formation showed that See1 actually acts in a cell-type specific manner: the small tumors induced by the *U. maydis see1* deletion mutant contain large hypertrophic (mesophyll derived) cells, but prominently lack the bundle sheath-derived hyperplasic cells, because the re-activation of cell cycle in these cells is depending on presence of See1 (Matei et al., under review). Although See1 is conserved in all other floral smuts, the vegetative organ-specific effectors such as See1 are only functional with the *U. maydis* native promoter, indicating their transcriptional regulation depends upon sensing of the target vegetative organ (Redkar et al., 2015b). This is a first system in fungal phyto-pathogens that shows the involvement of a specialized set of effectors in generation of abnormal cellular growth as infection symptoms.

## Metabolic Modulation by *U. maydis* in Tumor Formation

This part mainly highlights the physiological and metabolic changes that *U. maydis* largely tailors in young infected meristematic vegetative maize tissue towards its own benefits. At initial colonization stages between 1 and 2 dpi in expanding maize leaves, genes involved in light reactions, Calvin cycle, photorespiration, tetrapyrrole synthesis as well as sucrose and starch synthesis are not developmentally activated; as a consequence, the leaf is arrested and remains a sink rather than becoming a photosynthetically active source (Doehlemann et al., 2008; Kretschmer et al., 2016). Induction of sucrose degradation and reduction of sucrose synthesis was observed in infected tissues, indicating the import of sucrose from photosynthetically active source tissues. The tumor induction process induced by *U. maydis* generates a strong active sink. The tumorous tissue has increased free hexose, which is generated from the cleavage of imported sucrose and can be used by *U. maydis* as an easily accessible carbon source (Doehlemann et al., 2008). On the other hand, in order to achieve nutrient acquisition in the apoplast, the *U. maydis* plasma membrane transporter Srt1 allows direct utilization of sucrose without extracellular hydrolysis into monosaccharides (Wahl et al., 2010). This transporter, which outcompetes any known plant sucrose uptake system in substrate affinity, also offers a mechanism to prevent induction of plant defense responses known to occur upon apoplastic sucrose hydrolysis (Ehness et al., 1997; Kocal et al., 2008). A recent study has also shown the role of carbohydrate metabolism in tumor formation (Kretschmer et al., 2016). The authors found that injections of sucrose and glucose into the infection site stimulated virulence of *U. maydis* and led to a higher disease index, suggesting carbon is not only important but may also be limiting for tumor formation. Consequently, soluble carbohydrates in tumorous tissue is found to be similar to young sink leaf without active photosynthetic activity (Horst et al., 2008). Additionally, tumors are found to be influenced by carbon availability and sucrose signaling as addition of silver nitrate, which is known to interfere with the ethylene-dependent regulation of sugars, reduced tumor growth rate (Kretschmer et al., 2016). Along with the previously described transformation of source to sink tissue upon pathogen infection (Chandran et al., 2010) *U. maydis* tumor induction is also linked to a sink tissue induction in infected leaf areas as photosynthetic reactions are down regulated and photosynthetic pigments are degraded (Doehlemann et al., 2008; Horst et al., 2008; Kretschmer et al., 2016). Colonization of immature sink tissue interferes with the establishment of C4 photosynthesis in maize leaves, which results in lower CO_2_ assimilation (Horst et al., 2008). Although chloroplasts in tumorous tissue are retained, they redifferentiate into starch accumulating organelles (amyloplasts) inside the mesophyll altering the typical C4 dimorphism of starch accumulation primarily in the bundle sheath (Matei et al., under review). The tumors also represent a strong sink for organic nitrogen. Organic nitrogen accumulates in tumors at 8 dpi primarily during tumor expansion. The free amino acid pool is elevated in tumors during the entire infection process (Horst et al., 2010). On the other hand, metabolic modulation by *U. maydis* in floral tumors has not yet been investigated, and may hold surprises as floral organs are the strongest sinks on the plant.

## Conclusion and Perspective

In the last decade, numerous studies have identified cellular and metabolic modulation of the maize host by *U. maydis* in the course of infection and tumorigenesis. From comparison of the processes in floral and vegetative organs it is clear that *U. maydis* activates plant DNA synthesis and cell cycle in differentiated leaf cells and induces division in specific cell layers and cell types to form tumors. These diverse processes are programed by effector complexity in *U. maydis.* By evolution of leaf-specific effectors, this fungus gains an important host adaptation, one that is lacking in most relatives which are restricted to floral organs. By infecting leaves, *U. maydis* can accelerate its reproduction, an important factor in colonization of poaceae members at seedling stages. Infection of juvenile vegetative plant parts allows the fungus to complete its life cycle multiple times during plant vegetative growth and makes the infection cycle independent from the inflorescence development. Overall, tumors result from a complex process that involves immune suppression, nutrient re-channeling (modulation of cell cycle and organelle structure), sucrose and starch acquisition by generating an active sink, and uncontrolled host cell proliferation ultimately resulting in infectious symptoms by *U. maydis* (Doehlemann et al., 2008; Redkar et al., 2015a; Matei et al., under review). For a deeper understanding of these processes, cell-type specific effectors represent versatile molecular probes. Their functional characterization will provide crucial knowledge at the mechanistic level and therefore will be one of the major opportunities for future research.

## Author Contributions

AR did a substantial contribution to the design of this work outlay and important intellectual input in drafting of the work and final approval from all others. AM organized and prepared some of the parts of this review, gave intellectual inputs and contributed to designing of the model. AM also approved the writeup. GD contributed for writing and editing the major part of the review and was involved in approving the final version that is to be published.

## Conflict of Interest Statement

The authors declare that the research was conducted in the absence of any commercial or financial relationships that could be construed as a potential conflict of interest.
